# Exploring the Anti-Inflammatory Potential of *Ajuga integrifolia* Leaves Extract: In Vitro Dual Inhibition of Cyclooxygenase and Lipoxygenase Enzymes

**DOI:** 10.1155/2024/2938314

**Published:** 2024-10-28

**Authors:** Sisay Awoke Endalew, Belete Tesfaw Abebaw

**Affiliations:** Department of Chemistry, College of Natural Science, Wollo University, P.O. Box 1145, Dessie, Ethiopia

**Keywords:** *ajuga*, anti-inflammatory, COX, *integrifolia*, LOX

## Abstract

This study investigated the anti-inflammatory properties of *Ajuga integrifolia*, an herbal preparation. Qualitative and quantitative phytochemical analyses were conducted to identify active compounds in the preparation. The researchers also assessed its ability to inhibit the production of pro-inflammatory enzymes, cyclooxygenases (COX-1, COX-2), and lipoxygenase (5-LOX) in vitro. The extracts demonstrated dose-dependent inhibition of these enzymes, with some extracts showing IC_50_ values comparable to standard anti-inflammatory drugs. The ethanol extract exhibited significant inhibition of 5-LOX (52.99 μg/mL), compared to the standard drug zileuton (32.41 μg/mL), while the inhibition of COX-1 (66.00 μg/mL) and COX-2 (71.62 μg/mL) was comparable to the standard drug indomethacin (40.57 and 54.39 μg/mL, respectively). These findings suggest that *A. integrifolia* has the potential to be used as a herbal remedy for treating inflammatory conditions. By inhibiting pro-inflammatory enzymes, the extracts may effectively reduce inflammation and promote tissue healing or repair. The inhibition potential of extract of this plant can be taken as a good candidate of anti-inflammatory agent.

## 1. Introduction

Inflammation is a complex series of molecular reactions and cellular functions aimed at healing or repairing tissue [1–3]. This is a protective reaction of the body aimed at eliminating a harmful factor or limiting its spread, thereby eliminating neurotic cells and tissues [1]. Inflammatory agents may include infectious agents, immunological agents, physical agents, chemicals, and inert materials [4]. Persistent chronic inflammation is frequently linked to the development and advancement of several diseases, including cancer, arthritis, autoimmune disorders, cardiovascular issues, and neurological conditions [5–8]. The excessive buildup of substances involved in the arachidonic acid (AA) cascade, specifically the lipoxygenase (LOX) and cyclooxygenase (COX) pathways, is responsible for inflammatory diseases [9, 10], in which the mediators of the resulting inflammatory process are prostaglandins and leukotrienes, respectively [11].

COX-1 is constitutively expressed in most tissues and is involved in the production of prostaglandins that maintain physiological functions, such as protecting the gastric mucosa, regulating renal blood flow, and supporting platelet aggregation and normal blood clotting [9]. Inhibiting COX-1 can lead to therapeutic benefits, such as reducing pain and inflammation. COX-2 is typically induced during inflammation and is responsible for producing prostaglandins that mediate inflammatory responses, pain, and fever [12]. It is not usually present in significant amounts in healthy tissues. Inhibiting COX-2 is often desirable in therapeutic settings, particularly for managing pain and inflammation associated with conditions like arthritis, without the gastrointestinal side effects linked to COX-1 inhibition. Selective COX-2 inhibitors aim to provide anti-inflammatory benefits while sparing COX-1 activity [13, 14].

Various inflammatory diseases are often treated using nonsteroidal anti-inflammatory drugs (NSAIDs) [12, 15, 16]. Selective COX-2 inhibitors have been introduced to reduce NSAID-associated gastrointestinal toxicity [17]. Nevertheless, the use of these agents led to an elevated risk of cardiovascular complications, primarily attributed to reduced levels of prostaglandin I2 (PGI2) and increased levels of thromboxane A2 (TXA2) [18]. As a consequence of their adverse cardiovascular effects, certain inhibitors such as celecoxib and valdecoxib were withdrawn from the market [19]. Due to the side effects associated with commonly used NSAIDs such as indomethacin and zileuton, there remains a need to find safe and effective anti-inflammatory compounds from natural sources [20].

Herbal plant preparations have been used since prehistoric times to treat diseases and to treat many diseases, including inflammation, with medicinal plants around the world [14, 21–23]. The WHO estimates that four billion people use medicinal plants in some aspect of primary health care and that there is a growing trend toward natural treatments [24, 25]. Under these aspects, the medicinal properties of plants have been studied worldwide in terms of their strong biological activity in combating diseases, which do not cause side effects and are characterized by high economic viability [26–28]. According to Abebe, medicinal plants are widely used and attract great interest in Ethiopia due to their availability, acceptability, and biomedical well-being [29, 30].

*Ajuga integrifolia,* locally called *Armagusa* in Amharic, is a perennial plant that has many nutritive properties that can cure various diseases [31] such as anti-inflammatory [32–34], antiarthritic [35], antimalarial [36, 37], antibacterial [36, 38], antiplasmodial [39], antioxidant [40], gastrointestinal [41], hepatoprotective [42], anticancer [43], antifungal [44], antidiabetes, antihypertension, antiulcers, and dysuria [44, 45]. Even though it has been used as anti-inflammatory agents, there are no attempts that were made on COX-2 and 5-LOX dual inhibitors. Hence, the primary aim of this study was to assess the phytochemical characteristics, quantify phytochemical components, and investigate the in vitro dual inhibition of COX-2/5-LOX as an anti-inflammatory agent derived from leaves extracts of *A. integrifolia*.

## 2. Materials and Methods

### 2.1. Description of the Study Area

The sample of the plant material under the study was collected from Dessie which is located 400 km away from the capital city, Addis Ababa, Ethiopia. It has an elevation of 2131 m (6991 ft.) above sea level and latitude of 14°07′15.92″N and longitude of 38°43′24.13″E. The average annual temperature in Wollo is 18.3°C, and the average rainfall is 652 mm.

### 2.2. Plant Material Collection and Authentication

The fresh leaves of the plant were collected from Tamira Ber (Debre Sina), in the month of January 2022 following the guidelines proposed by Wondafrash [46]. The plant materials were collected after obtaining written consent from the local authority and a special letter from the Wollo University Postgraduate Office. The plant specimen was submitted to Wollo University Herbarium Center for identification and was identified as *A. integrifolia* by Belay Mesele botanist of the Department of Biology, Wollo University.

### 2.3. Chemicals

Chemicals used in this study were diethyl ether (DEE), chloroform (CHCl_3_), ethyl acetate (EtOAc), ethanol (EtOH), methanol (MeOH), ferric chloride (FeCl_3_), copper sulfates (CuSO_4_), sodium nitroprusside, acetic anhydride, aluminum chloride (AlCl_3_), sulfuric acid (H_2_SO_4_), dimethyl sulphoxide (DMSO), sodium nitrous (NaNO_2_), sodium carbonate (Na_2_CO_3_), concentrated hydrochloric acid (HCl), sodium hydroxide (NaOH), sodium potassium tartrate, ferric chloride hexahydrate (FeCL_3_.6H_2_O), lead tetra acetate (Pb(C_2_H_3_O_2_)_4_), concentrated nitric acid (HNO_3_), and glacial acetic acid (CH_3_COOH). All chemicals and reagents used in this study were imported from CDH P. Ltd., New Delhi, India (ISO 9001).

### 2.4. Preparation of the Extracts

The leaves were dried at a temperature of 25°C for a period of 10 days, taking care to avoid exposure to sunlight. This drying step helps to remove excess moisture from the leaves and prepare them for the extraction process. The dried leaves were ground into a powder using an electric mortar. This step increases the surface area of the plant material, facilitating better extraction of the desired compounds. The powdered plant material was stored in an airtight container, ensuring the preservation of its quality until further extraction. The pulverized plant material was subjected to sequential extraction using solvents of increasing polarity. This approach allows for the extraction of compounds with different polarities, covering a wide range of extracted compounds.

To determine the qualitative composition of the extracts, they were placed in preweighed vials, which enabled later analysis and characterization of the extracted compounds. Approximately 300 g, measured by electrical beam balances (1810-BA Model, Beijing, China) of the sample, was mixed with 1000 mL of DEE in a 1500 mL Erlenmeyer flask. The mixture was then placed on a shaker for 16 h, allowing for efficient extraction. The extract was separated from the residue using a separating funnel and then concentrated using a rotary evaporator (model: YC7124, Beijing, China). This concentration step helps to remove excess solvent and obtain a more concentrated extract. The remaining residue from the previous extraction step was subjected to sequential extraction using 1000 mL of chloroform, with stirring for 16 h. Similarly, the residue was also extracted using ethyl acetate and ethanol, following the same procedure. These additional extractions with different solvents further enhance the range of compounds extracted.

### 2.5. Qualitative Phytochemical Estimation of Extracts

Qualitative phytochemical analysis of extracts to identify alkaloids, saponins, tannins, flavonoids, steroids, glycosides, phenolics, terpenoids, carbohydrates, and proteins following standard procedures [47–49].

### 2.6. Quantitative Phytochemical Estimation

#### 2.6.1. Estimation of Total Phenolic Content (TPC)

Folin–Ciocalteu reagent with gallic acid as standard was used to determine the amount of TPC in the extracts [50, 51] and was expressed in mg/g gallic acid equivalent (GAE/g). Various concentrations of gallic acid (20, 40, 60, 80, and 100 μg/mL) in methanol were prepared. Furthermore, a plant extract at a concentration of 100 μg/mL in MeOH was also prepared. Each sample (0.5 mL) was combined with 2.5 mL of Folin–Ciocalteu reagent and thoroughly mixed. The mixture was left at room temperature for 30 min and the absorbance was subsequently measured at 760 nm using a UV–Vis spectrophotometer (T60 UV-VS spectrophotometer). The reagent reflects the reducing compounds and gives the reaction a blue color.

#### 2.6.2. Estimation of Total Flavonoid Content (TFC)

The TFC was determined by the AlCl_3_ method using rutin as a standard solution. Rutin with a concentration of 20–100 μg/mL was prepared in methanol [52]. A solution of the sample (0.5 mL) at a concentration of 100 μg/mL in MeOH was combined with 2 mL of distilled water. To this mixture, 0.15 mL of a 5% NaNO_2_ solution was added, and the solution was left undisturbed for 6 min. Following that, 0.15 mL of a 10% AlCl_3_ solution was introduced and allowed to stand for an additional 6 mins. Finally, 2 mL of a 4% NaOH solution was added to the resulting mixture [53]. Water was immediately added to bring the final volume to 5 mL, and the mixture was mixed thoroughly and allowed to stand for an additional 15 mins. The absorbance of the mixture was measured at 510 nm using water as a blank and the TFC was expressed as mg RE/g extracts.

#### 2.6.3. Estimation of Total Alkaloids Content (TAC)

A hundred micrograms per milliliter of plant extract was prepared by dissolving in DMSO, adding 1 mL 2N HCl, and filtering. Bromocresol green solution and phosphate buffer (5 mL each) were added to this solution in a separatory funnel [51]. The mixture was stirred vigorously with a gradual addition of CHCl_3_ and collected in a 10 mL volumetric flask until the mark was reached. Standard reference solutions of atropine were prepared with a concentration ranging from 20 to 100 μg/mL [54]. The absorbances were measured against the blank reagent at 470 nm and the total alkaloid content was expressed as mg of AE/g of extract.

#### 2.6.4. Estimation of Total Saponins Content (TSC)

TSC was determined by the method described by Kamani, et al. based on vanillin sulfuric acid colorimetric reaction with some modifications [55]. Five different concentrations (20–100 μg/mL) of standard diosgenin solution were prepared. About 50 μL of plant extract was dissolved with 250 μL of distilled water and 250 μL of vanillin reagent was added. Subsequently, the solution was placed in a water bath at 60°C for a duration of 10 min and then rapidly cooled using ice-cold water. The absorbance of the solution was measured at 544 nm. The obtained values were expressed as DE mg/g extracts using a standard curve. All the quantitative measurements were conducted in triplicate.

## 3. Enzyme Assays

### 3.1. COX-1 and COX Two Inhibitory Assay

In vitro COXs inhibitory activities of crude extract of *A. integrifolia* were measured using COX inhibitor screening kit from Cayman Chemical Company (Ovine/Human, Cayman Chemical Company, Ann Arbor, MI) with 96-well plates [56]. The peroxidase activity, which is an indicator of COX activity, was measured calorimetrically. The presence of oxidized N,N,N′,N′-tetramethyl-*para*-phenylene-diamine (TMPD) was monitored at 590 nm [57]. For the assay, in the reaction medium, the test enzyme and inhibition test batches consisted of assay buffer (150 μL), heme, enzymes, and DMSO (10 μL each) and dilutions of each test extract (20, 40, 60, 80 and 100 μg/mL) and same concentrations of indomethacin, respectively. The blank also consisted of 150 μL of assay buffer. The mixture was briefly shaken and then incubated at 25°C for 5 min. Subsequently, 20 μL of colorimetric substrate was added to each well, followed by the addition of 20 μL of AA solution to all the wells. The plate was gently dried for a few seconds to ensure thorough mixing and then incubated at 25°C for 2 min. All tests were carried out in triplicate. The absorbance was read at 590 nm with a plate reader and the percent inhibition (PI) was calculated using the following formula and plotted with the PI against the inhibitor concentration to determine the IC_50_ value [13]. The PI values were plotted against the inhibitor concentration to determine the IC_50_ value, which represents the concentration of the inhibitor required to inhibit 50% of the enzyme activity.

(1) $$\begin{array}{c} \%\text{ Inhibition of cyclooxygenase}=\frac{\left(\text{control}-\text{test sample}\right)}{\text{control}}\times 100, \end{array}$$

where control is an enzymatic activity without inhibitor and sample is an enzymatic activity in the presence of extracts or reference compound.

### 3.2. 5-LOX Inhibitory Assay

The inhibition of LOX was evaluated according to the method described by Malterud [58] with slight modifications. A 100 μL enzyme solution was prepared in boric acid buffer (0.2 M; pH 9.0). To assess the inhibitory effect, 25 μL of each test extract (20, 40, 60, 80, and 100 μg/mL) was added to the enzyme solution. The mixture was then incubated at room temperature for 3 min.

The reaction was then initiated by the addition of 125 μL of the substrate (250 μM of linoleic acid) and the velocity was recorded for 3 min at 234 nm with a plate reader. Zileuton was used as a reference compound. The percentage of LOX inhibition was calculated according to the following equation [13]:

(2) $$\begin{array}{c} \%\text{ inhibition of lipooxygenase}=\frac{\left(\text{control}-\text{test sample}\right)}{\text{control}}\times 100. \end{array}$$

### 3.3. Statistical Analysis

The PI measurements of COX-1, COX-2, and 5-LOX were obtained with at least three replicates. IC_50_ values were calculated by regression analysis using Microsoft Excel involving five concentrations. The percentage inhibition of enzyme activity and IC_50_ values are presented as the mean values ± SD. Statistical significance among the samples was analyzed using SPSS (IBM SPSS Statistics 20) and Microsoft Excel 2016 (KB4011684).

## 4. Results and Discussions

Water-based methods, such as decoction and infusion, are traditionally used and generally safer, effectively extracting hydrophilic compounds while preserving their biological activity. However, they may yield lower amounts of non-polar compounds and can degrade heat-sensitive molecules. In contrast, solvent-based methods can extract a broader range of compounds and typically provide higher yields, particularly for lipophilic substances. The choice of extraction method should align with the target compounds and the research objectives, emphasizing the need for optimized conditions that balance yield, purity, and safety [59].

### 4.1. Qualitative Phytochemical Screening

As shown in Table 1, tests regarding the presence or absence of phytoconstituents in the leaves of *A. integrifolia* extracts of DEE, CHCl_3_, EtOAc, and EtOH using different types of test methods have been assessed. Thus, the result revealed that flavonoids and glycosides were detected in all extracts. All tested phytochemicals are detected in EtOH extract except saponins. The result obtained in this study is in agreement with previously reported data [60, 61].

### 4.2. Quantitative Phytochemical Analysis of Crude Extracts

The quantitative analysis of some phytochemicals in the extract has been evaluated and tabulated as shown in Table 2. The TPC, TFC, alkaloids, and TSC of different extracts of the plant material were estimated equivalent to gallic acid, rutin, atropine, and diosgenin solutions, respectively, at the concentration of 20, 40, 60, 80, and 100 μg/mg. The linear regression curve indicated that all are in the highly acceptable range [31].

As shown in Table 3, the phenolic inhibitory activities were 0.1089, 0.1751, 0.2320, 0.2776, and 0.3250 at 20, 40, 60, 80, and 100 μg/mg solution of gallic acid, respectively. The TPC in the concentrations of DEE, CHCl_3_, EtOAc, and EtOH of extracts were estimated as 9.0, 63.0, 54.5, and 182.5 mg/mgGA equivalent with mean absorbance of 0.081 ± 0.002, 0.189 ± 0.001, 0.172 ± 0.004, and 0.428 ± 0.002. The flavonoid inhibitory activities were 0.112, 0.142, 0.167, 0.183, and 0.202 at the same concentration solution of rutin.

The TFCs DEE, CHCl_3_, EtOAc, and EtOH were 37.0, 95.0, 138.0, and 165.0 mg/mgRE which was equivalent to Rutin with the mean of absorbance was 0.131 ± 0.004, 0.189 ± 0.002, 0.232 ±0 .002, and 0.259 ± 0.005, respectively.

**Figure 1:**
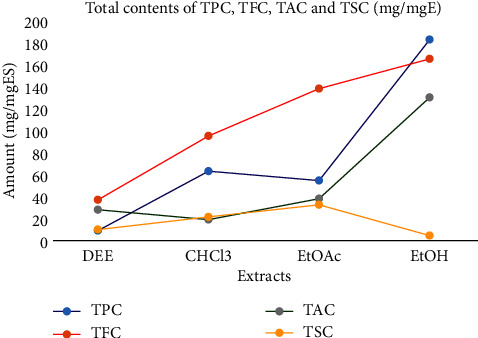
Total contents of phenols, flavonoids, alkaloids, and saponins.

The alkaloid inhibitory activities were recorded as 0.1089, 0.1751, 0.232, 0.2776, and 0.3251 in atropine solution. The total alkaloid content was measured as 28.0, 19.0, 38.0, and 130.0 mg/mgA with mean absorbance of 0.103 ± 0.002, 0.094 ± 0.002, 0.113 ± 0.002, and 0.205 ± 0.004 for DEE, CHCl_3_, EtOAc, and EtOH, respectively. The saponins inhibitory activities were 0.159, 0.192, 0.235, 0.304, and 0.353 mg/mgD in the diosgenin solution. The total saponins content was detected as 10.0, 21.5, 32.5, and 4.5 for four extracts in the same order with 0.118 ± 0.003, 0.141 ± 0.003, 0.163 ± 0.002, and 0.107 ± 0.001 of mean absorption. As depicted in Figure 1, the TPC and TFC are higher in EtOH extract; however, relatively flavonoids are high in DEE, CHCl_3_, and EtOAc extracts as compared with TPC, TAC, and TSC.

### 4.3. Inhibitory Activities of Crude Extracts

**Figure 2:**
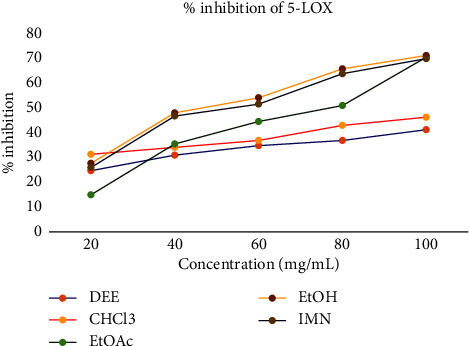
Percent inhibition of crude extracts against 5-LOX enzyme.

As shown in Table 4 and Figure 2, the highest 5-LOX inhibitory activities of DEE, CHCl_3_, EtOAc, and EtOH extracts at the concentrations of 20–100 μg/mL were found to be 41.51, 46.58, 70.93, and 71.63, respectively, with IC_50_ value of 141.9, 118.76, 70.16, and 52.99.

**Figure 3:**
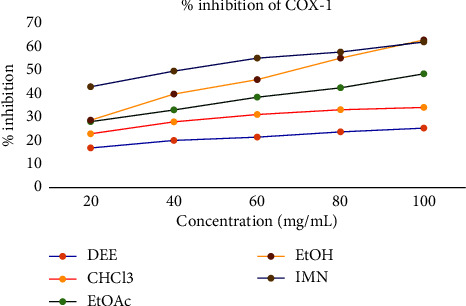
Percent inhibition potential of crude extracts against COX-1.

Table 4 and Figure 3 show that the COX-1 inhibitory activities of the extracts ranged from 25.89% to 64.21% for DEE, CHCl3, EtOAc, and EtOH, respectively, at a concentration of 100 mg/mL. Notably, the EtOH extract exhibited higher inhibitory activity than the other extracts at each concentration. The inhibiting activities of those extracts against COX-2 were recorded in the range of 43.87%–69.48%. The IC_50_ value of crude extracts against COX-1 was measured as 330.00, 199.57, 103.24, and 66.00 with increasing polarity. The lowest IC_50_ value corresponds with the highest inhibitory activity of the extract. Relatively, EtOH extract has a higher efficacy than the rest crude extracts.

**Figure 4:**
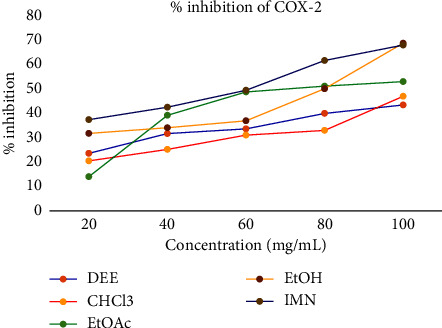
Percent inhibition potential of crude extract against COX-2.

As shown in Table 4 and Figure 4, the inhibitory activity of each extract toward COX-2 was evaluated as 43.87, 47.46, 53.55, and 69.48 at 100 mg/mL solution. Its IC_50_ value recorded was 122.93, 119.77, 78.524, and 71.62 μg/mL, respectively, for DEE, CHCl3, EtOAc, and EtOH.

Based on the provided Fable 3 5, IC_50_ values were determined for different test solutions against the tested enzymes (5-LOX, COX-1, and COX-2). The IC_50_ value represents the concentration of a test solution required to inhibit the enzyme activity by 50%. Table 4 mentions that the least inhibition of COX-1 and highest IC_50_ values were observed for the DEE solvent with an IC_50_ value of 330.00 μg/mL. On the other hand, the highest inhibition of COX-1 and lowest IC_50_ values were registered for EtOH (ethanol) with an IC_50_ value of 66.00 μg/mL. This indicates that EtOH has a stronger inhibitory effect on COX-1 compared to the DEE solvent. Similarly, for COX-2 inhibition, the least inhibition and highest IC_50_ values were observed for the DEE extract with an IC_50_ value of 122.93 μg/mL, while the highest inhibition and lowest IC_50_ values were registered for EtOH with an IC_50_ value of 71.62 μg/mL.

This suggests that EtOH exhibits stronger inhibitory activity against COX-2 compared to the DEE extract. Moving on to 5-LOX inhibition, the document states that the least inhibition and highest IC_50_ values were recorded for DEE with an IC_50_ value of 141.9 μg/mL, whereas high inhibition and a low IC_50_ value of 52.99 μg/mL were measured for EtOH. This implies that EtOH has a more potent inhibitory effect on 5-LOX compared to the DEE extract. In summary, the data presented in the document indicate that EtOH demonstrates stronger inhibitory activity against all three enzymes (COX-1, COX-2, and 5-LOX) compared to the DEE extract. This suggests that EtOH contains bioactive compounds that have potential therapeutic relevance in modulating the activity of these enzymes as confirmed in Table 1. However, further studies are required to identify and characterize the specific compounds responsible for these inhibitory effects and to evaluate their potential clinical applications.

The relationship between phytochemicals in plant extracts and their anti-inflammatory activity has been the subject of extensive research. The presence of those potential compounds in the extract as confirmed by Table 1 was indicative of the promising result recorded in this study. The presence of phenolic compounds, such as flavonoids and phenolic acids, has been recognized for their anti-inflammatory properties. Flavonoids, such as quercetin, kaempferol, and apigenin, have shown inhibitory effects on various pro-inflammatory mediators, including cytokines, chemokines, and enzymes such as COX and LOX [28]. Phenolic acids, such as rosmarinic acid and caffeic acid, have also exhibited anti-inflammatory effects by modulating inflammatory pathways [62]. Alkaloids are another group of phytochemicals found in *A. integrifolia* plant extracts that have demonstrated anti-inflammatory activity. Various alkaloids, such as berberine, curcumin, and reserpine, have shown inhibitory effects on pro-inflammatory cytokines and enzymes involved in inflammation [63]. These alkaloids can suppress the production of inflammatory mediators and regulate immune responses.

Terpenoids, such as curcumin, gingerol, and boswellic acid, have exhibited anti-inflammatory effects by inhibiting pro-inflammatory enzymes (COX and LOX) and modulating inflammatory signaling pathways [64]. These compounds have shown potential in reducing inflammation and alleviating inflammatory diseases. Saponins and glycosides, the other groups of compounds, are responsible for the potent anti-inflammatory effect of plant extract. They can inhibit the production of inflammatory cytokines, such as tumor necrosis factor-alpha (TNF-*α*) and interleukins (ILs), and suppress the activation of inflammatory pathways [65]. Saponins, such as ginsenosides and glycyrrhizin, have been reported to possess significant anti-inflammatory effects in both in vitro and in vivo studies. The anti-inflammatory activity of these phytochemicals is often attributed to their ability to modulate key inflammatory pathways, including the NF-*κ*B (nuclear factor kappa-light-chain-enhancer of activated B cells) pathway, MAPK (mitogen-activated protein kinase) pathway, and inflammasome activation [66]. These compounds can interfere with the production of pro-inflammatory mediators, inhibit the expression of inflammatory genes, and regulate immune responses, thereby reducing inflammation.

The significant anti-inflammatory properties *A. integrifolia* have been reported. Shin et al. demonstrated the inhibitory effect of extract on pro-inflammatory mediators such as nitric oxide (NO) and TNF-*α* in vitro [28]. The extract exhibited a dose-dependent reduction in the production of these inflammatory markers. Other species in the genus such as *Ajuga reptans* has been studied for its anti-inflammatory potential by inhibiting the production of pro-inflammatory cytokines and mediators, such as TNF-*α*, ILs, and COX enzymes [31]. *Ajuga bracteosa* is another species within the Ajuga genus that has been investigated for its anti-inflammatory activity [32], and *Ajuga chamaepitys* has been traditionally used for its medicinal properties, including anti-inflammatory effects inhibiting the production of inflammatory cytokines and enzymes [67].

The enzyme assay confirmed that *A. integrifolia* exhibits inhibitory effects on COX and LOX, which are key enzymes involved in the inflammatory process. Inhibition of these enzymes can reduce the production of pro-inflammatory mediators such as prostaglandins and leukotrienes. This observation is clinically relevant because it suggests that *A. integrifolia* may have potential as a natural source for developing anti-inflammatory agents targeting COX and LOX pathways. Currently, NSAIDs are commonly prescribed to treat inflammation. However, long-term use of NSAIDs can lead to adverse effects on the gastrointestinal system, cardiovascular health, and kidney function. The discovery of *A. integrifolia*'s inhibitory effects on COX and LOX provides an alternative approach to developing anti-inflammatory agents that may have a reduced risk of such side effects. This could be of great clinical relevance, especially for patients who require long-term anti-inflammatory therapy. In summary, the discovery of *A. integrifolia's* anti-inflammatory effects, specifically targeting COX and LOX, has clinical relevance by providing a potential natural alternative to NSAIDs. It opens up opportunities for further research can lead to the development of novel anti-inflammatory agents with improved efficacy and reduced side effects.

## 5. Conclusion

The development of dual 5-LOX/COX inhibitors is currently a unique approach for developing compounds with enhanced anti-inflammatory effects and attenuated cardiovascular and gastrointestinal side effects. The EtOAc fraction of the plant leaves extract showed good potential for inhibiting both COX-1/2 and 5-LOX. Among all the extracts, EtOH showed that excellent activity against COX-1, COX-2, and 5-LOX enzyme with observed IC_50_ values was 66.00, 71.62, and 52.99 μg/mL, respectively. The overall results revealed that the significant anti-inflammatory activity can be raised from high content of highly polar phenols, flavonoids, and alkaloids.

The study highlights the anti-inflammatory potential of *A. integrifolia*, attributing its effects to various phytochemicals present in the plant extract. These compounds, including flavonoids, phenolic acids, alkaloids, terpenoids, saponins, and glycosides, have demonstrated inhibitory effects on pro-inflammatory mediators and modulation of inflammatory pathways. The extract showed inhibitory effects on COX and LOX enzymes, suggesting its potential as a natural source for developing anti-inflammatory agents. This discovery presents an alternative to NSAIDs with potentially reduced side effects. However, the specific mechanisms through which the phytochemicals exert their anti-inflammatory effects remain unexplored, although a comprehensive chemical characterization of the extracts is necessary to identify and quantify the specific compounds responsible for the observed activity. The author suggests several areas for further investigation to enhance the completeness of the manuscript, including in vivo studies, exploration of the mechanism of action, chemical characterization, comparative studies, toxicological evaluation, and pharmacokinetic studies.

## Figures and Tables

**Table 1:** Phytochemical analysis of crude extracts.

Phytochemicals	Types of tests	Observation			
		Diethyl ether	CHCl_3_	EtOAc	EtOH
Carbohydrates	Molisch test	−	−	+	+
Proteins	Ninhydrin test	−	+	+	+
Alkaloids	Hager's test	−	−	−	+
Terpenoids	Salkowski test	−	−	+	+
Saponins	Froth test	−	+	+	−
Phenolics	Lead acetate test	−	+	+	+
Flavonoids	Shinoda test	+	+	+	+
Glycoside	Keller-Killiani test	+	+	+	+

**Table 2:** Percent inhibition of standards (gallic acid, rutin, atropine, and diosgenin).

Concentration (μg/mg)	% inhibition			
	Galic acid (GA)	Rutin (R)	Atropine (A)	Diosgenin (D)
20	0.1089	0.1120	0.1210	0.1590
40	0.1751	0.1420	0.1420	0.1920
60	0.2320	0.1670	0.1960	0.2350
80	0.2776	0.1830	0.2380	0.3040
100	0.3251	0.2020	0.2730	0.3530
*Y* = *ax* + *b*	*Y* = 0.002*x* + 0.063	*Y* = 0.001*x* + 0.094	*Y* = 0.002*x* + 0.063	*Y* = 0.002*x* + 0.098
Linear reg. (*R*^2^)	0.9935670	0.9841420	0.9868015	0.9848258

**Table 3:** Total phenol, flavonoid, alkaloid, and saponins content.

Constituents	Expressed values (mg/mg)	Mean absorbance and total amount of constituents			
		DEE	CHCl_3_	EtOAc	EtOH
TPC	Mean	0.081 ± 0.002	0.189 ± 0.001	0.172 ± 0.004	0.428 ± 0.002
	mg/mgGA	9.0	63.0	54.5	182.5

TFC	Mean	0.131 ± 0.004	0.189 ± 0.002	0.232 ± 0.002	0.259 ± 0.005
	mg/mgR	37.0	95.0	138.0	165.0

TAC	Mean	0.103 ± 0.002	0.094 ± 0.002	0.113 ± 0.002	0.205 ± 0.004
	mg/mgA	28.0	19.0	38.0	130.0

TSC	Mean	0.118 ± 0.003	0.141 ± 0.003	0.163 ± 0.002	0.107 ± 0.001
	mg/mgD	10.0	21.5	32.5	4.5

**Table 4:** 5-LOX, COX-1, and COX-2 inhibitory activities of extracts.

Extracts/standards	Concentration (in μg/mL)	% inhibition			IC_50_ value (in μg/mL)		
		5-LOX	COX-1	COX-2	5-LOX	COX-1	COX-2
DEE	20	24.87	17.26	23.81	141.9	330.00	122.93
	40	31.17	20.53	32.01			
	60	35.03	21.98	33.96			
	80	37.13	24.25	40.36			
	100	41.51	25.89	43.87			

CHCl_3_	20	31.52	23.43	20.69	118.76	199.57	119.77
	40	34.33	28.61	25.45			
	60	37.13	31.79	31.38			
	80	43.26	33.88	33.33			
	100	46.58	34.88	47.46			

EtOAc	20	15.06	28.70	14.13	70.16	103.24	78.524
	40	35.73	33.79	39.58			
	60	44.83	39.33	49.26			
	80	51.31	43.42	51.68			
	100	70.93	49.50	53.55			

EtOH	20	27.85	29.34	32.08	52.99	66.00	71.62
	40	48.34	40.69	34.43			
	60	54.47	46.96	37.31			
	80	66.20	56.31	50.59			
	100	71.63	64.21	69.48			

Zileuton (5-LOX) Indomethacin (COX-1/2)	20	26.14	43.87	37.78	32.41	40.51	54.39
	40	47.05	50.68	42.94			
	60	51.89	56.31	49.96			
	80	64.23	58.95	62.30			
	100	70.36	63.31	68.70			

## Data Availability

Data are available on request from authors.
